# Isolated Partial Absence of the Septum Pellucidum: A Case Report

**DOI:** 10.7759/cureus.67604

**Published:** 2024-08-23

**Authors:** Sanjay M Khaladkar, Sravya Julakanti, Sayali Paidlewar, Ankita Pandey

**Affiliations:** 1 Radiodiagnosis, Dr. D. Y. Patil Medical College, Hospital and Research Centre, Dr. D. Y. Patil Vidyapeeth (Deemed to be University), Pune, IND

**Keywords:** commissure, absence, neuroimaging, fornix, seizures, septum pellucidum

## Abstract

The septum pellucidum is an important thin, membranous structure in the brain that separates the anterior horns of the lateral ventricles, essential for maintaining brain anatomy and function. Here, we describe a case of a 38-year-old male with a 20-year history of seizures, occurring approximately three to four times annually and lasting 30 minutes to one hour per episode, who presented with a recent seizure three days prior. Magnetic resonance imaging (MRI) of the brain revealed an absence of the septum pellucidum in its posterior portion, mild prominence of both lateral ventricles, and an abnormal course of the crura of the fornix, leading to a diagnosis of partial absence of the septum pellucidum. This case underscores the importance of comprehensive neuroimaging in detecting structural brain anomalies, which is crucial for effective diagnosis, management, and improving patient outcomes, particularly in long-standing seizure disorders.

## Introduction

The septum pellucidum is a thin, membranous structure in the brain that separates the anterior horns and body of the lateral ventricles [1]. This structure plays a critical role in maintaining the normal anatomy and function of the brain. The septum pellucidum forms a crucial part of the brain's ventricular system, which is involved in the production and circulation of cerebrospinal fluid [2]. Its integrity is necessary for the proper functioning of this system, as well as for the structural stability of surrounding brain tissues. Given its central location and functional significance, any abnormalities in the septum pellucidum can have widespread effects on brain physiology and neural activity [3,4].

Absence or malformation of the septum pellucidum can be associated with a range of neurological conditions. These conditions include seizures, which are characterized by abnormal electrical activity in the brain, and developmental delays, which can affect cognitive, motor, and social skills [5]. Other cognitive impairments associated with anomalies in the septum pellucidum can range from mild learning disabilities to more severe intellectual disabilities [6]. Clinicians need to be aware of the implications of anomalies in the septum pellucidum to diagnose and manage patients effectively.

Partial or complete absence of the septum pellucidum occurs in two or three individuals per 1,00,000 general population [7].

## Case presentation

A 38-year-old male presented with a history of seizures, the most recent occurring three days prior to the presentation. The patient was experiencing seizures for the past 20 years and was on antiseizure medication. The patient was on levetiracetam 500 mg twice a day and carbamazepine 300 mg twice a day. Each episode ranged between 30 minutes to one hour with a frequency of approximately three to four times a year.

During the seizures, the patient experienced upward rolling of the eyes and generalized tonic-clonic convulsions with drooling of saliva. The patient did not report any history of postictal confusion, headache, weakness or loss of power in the limbs, trauma, ear or nose or throat bleeding, dizziness, altered sensorium or loss of consciousness, slurred speech, facial deviation, visual or hearing disturbances, or bladder or bowel dysfunction. The patient's general condition was unremarkable, and there were no focal neurological deficits noted upon examination.

**Figure 1:**
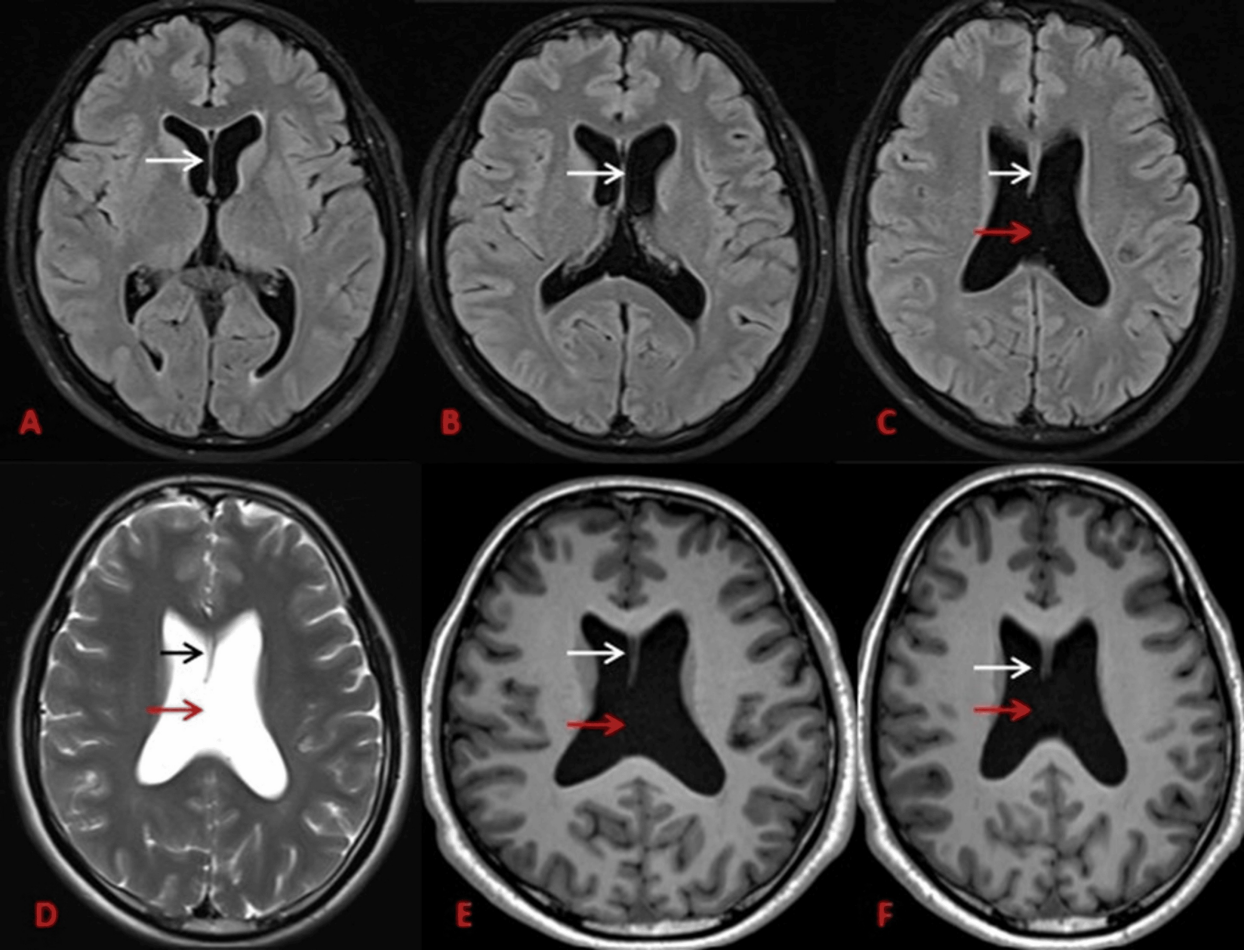
MRI brain (Axial): A-F showing the anterior portion of the septum pellucidum in A-C-FLAIR, D-T2WI, and E-F-T1WI (marked by black and white arrows) and absence of the posterior portion of the septum pellucidum (C-F) (marked by the red arrow). FLAIR: fluid-attenuated inversion recovery, T2WI: T2-weighted imaging, T1WI: T1-weighted imaging

**Figure 2:**
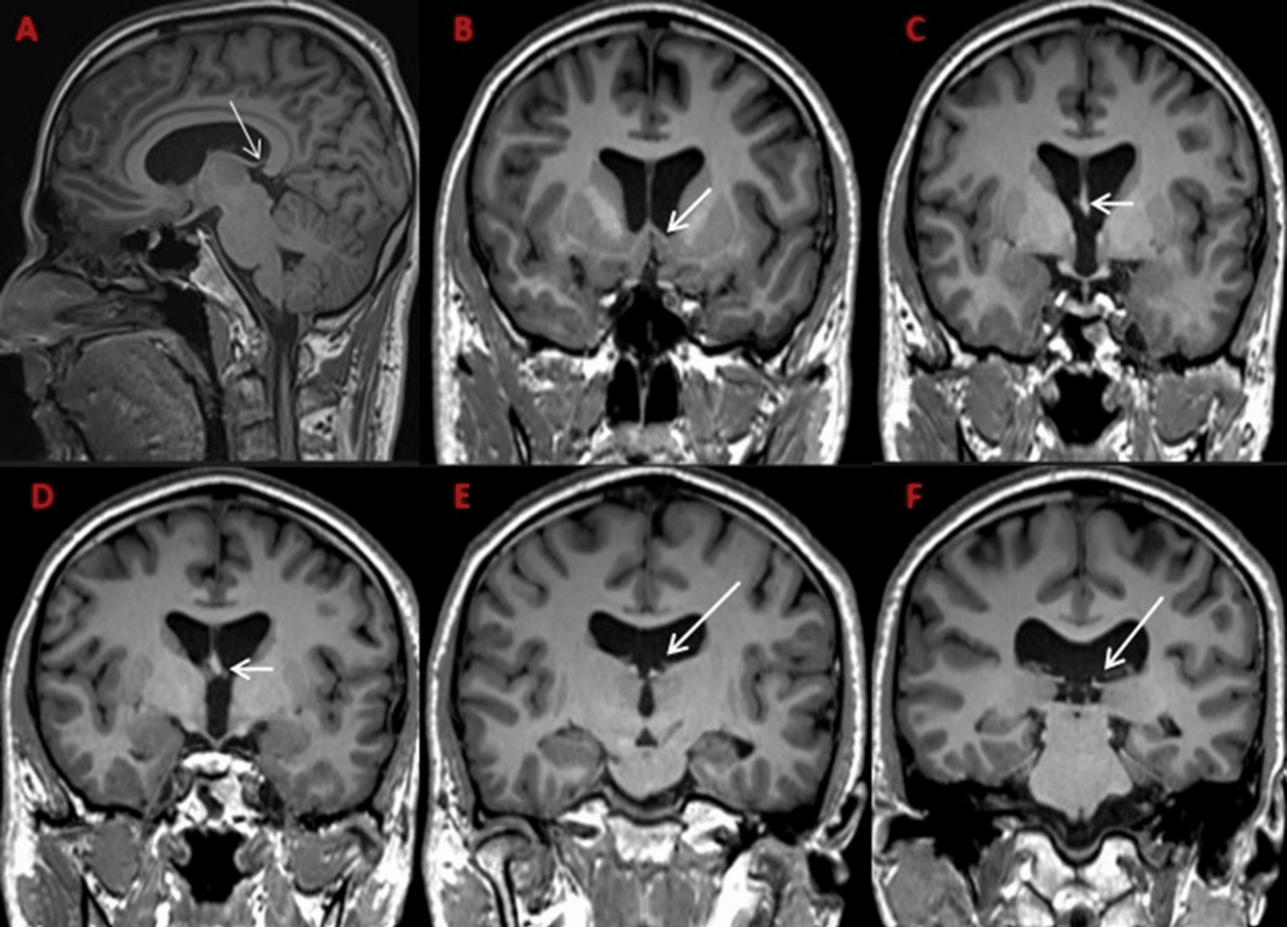
Abnormal course of the posterior portion of the body and crura of the fornix in a partial absence of the septum pellucidum A: sagittal T1 showing the inferiorly placed crura and posterior portion of the body of the fornix (marked by the white arrow). B: coronal T1 showing normal columns of the fornix (marked by the white arrow). C, D: coronal T1 showing the normal body of the fornix (marked by the white arrow). E, F: coronal T1 showing the inferiorly placed crura of the fornix due to the absence of the posterior portion of the septum pellucidum (marked by the white arrow).

History of diabetes mellitus (DM), hypertension (HTN), tuberculosis (TB), asthma, ischemic heart disease (IHD), relevant surgeries, or addictions were evaluated and found negative. Magnetic resonance imaging (MRI) of the brain revealed the absence of the septum pellucidum in its posterior portion (Figure 1). There was mild prominence of both lateral ventricles, while the third and fourth ventricles appeared normal. The crura of the fornix exhibited an abnormal course, with the crura and posterior portion of the body positioned inferiorly. There was no observed connection between the two crura or formation of a commissure. The anterior portion of the body and columns of the fornix appeared normal (Figure 2). No other anomalies were detected. The diagnosis of partial absence of the septum pellucidum was made based on MRI findings.

## Discussion

The septum pellucidum is a crucial but relatively understudied structure in the brain. The septum pellucidum comprises two thin, transparent layers that stretch from the front section of the body, known as the genu, and the rostrum of the corpus callosum to the upper surface of the fornix. This structure connects the corpus callosum above with the fornix below, spanning the midline gap. The ventricular side of the septum pellucidum is coated with ependymal lining. The septum pellucidum is a relay station, with its primary anatomical and functional fiber connections being to the hippocampus and the hypothalamus rather than the primary olfactory structures [4,8].

The fornix is anatomically divided into segments, including the crura, commissure, body, and columns, extending from posterior to anterior. Typically, the crura curve is in an antero-superior direction beneath the splenium of the corpus callosum, with one for each hemisphere. These crura meet at the midline to form the commissure, which serves as a bridge between opposite sides. Continuing beyond the commissure, the crura merge to form the body, which attaches to the underside of the septum pellucidum. At the level of the foramen of Monroe, the body divides into two columns that bend downward toward the anterior commissure [9].

The fornix is a curved bundle of fibers responsible for transmitting signals from the hippocampus to the mammillary bodies and septal nuclei and appears as C or arch-shaped on sagittal magnetic resonance imaging of the brain. The inferior border of the septum pellucidum is connected to the upper margin of the forniceal body. Hence, in the absence of septum pellucidum due to inadequate support from septum pellucidum, fornix is displaced inferiorly with loss of normal C or arch shape in its posterior portion. Inferiorly displaced fornix can occasionally obstruct the foramen of Monroe with resultant obstructive hydrocephalus [10].

The septum pellucidum's formation is closely intertwined with the development of the corpus callosum and the two other forebrain commissures. The absence of the septum pellucidum is associated with other malformations, such as septo-optic dysplasia, schizencephaly, Chiari II malformation, holoprosencephaly, encephalocele, agenesis of the corpus callosum, porencephaly, hydranencephaly, and hydrocephalus with aqueductal stenosis, in each instance [11].

The condition is linked to various neurological conditions, including seizures, developmental delays, and other cognitive impairments [5,12,13]. Patients with these anomalies may present with a spectrum of symptoms, ranging from mild to severe, affecting their cognitive, motor, and social skills [5,14]. In the presented case, the patient's seizures without other significant neurological deficits suggest that the septum pellucidum anomaly might be an isolated finding rather than part of a broader syndrome. However, the potential for other undetected anomalies underscores the importance of comprehensive neurological evaluations [15].

Magnetic resonance imaging (MRI) is a useful imaging modality in detecting the complete or partial absence of septum pellucidum. These imaging modalities not only confirm the presence of structural abnormalities but also help clinicians assess the implications for brain function [16]. Detailed imaging can reveal associated abnormalities in surrounding structures, which is crucial for determining the appropriate clinical approach. In this case, the MRI findings of an abnormal course of the crura of the fornix and the absence of a commissure between the two crura highlighted the complexity of the anomaly. Such detailed imaging aids in tailoring therapeutic strategies, which may include antiepileptic medications, cognitive therapy, and monitoring for potential developmental issues [1].

Early neuroimaging is aimed at the detection of complete or partial absence of septum pellucidum, associated anomalies (like agenesis of the corpus callosum, septo-optic dysplasia, schizenencephaly, Arnold-Chiari malformation, and holoprosencephaly). Isolated absence of the septum pellucidum usually has a favorable developmental prognosis. However, neurology, endocrinology, and ophthalmological evaluations are needed for follow-up examinations to detect early symptoms or signs of associated anomalies [7].

## Conclusions

Agenesis of the septum pellucidum can be complete or partial. Usually, it is associated with other conditions like agenesis of the corpus callosum, septo-optic dysplasia, holoprosencephaly, or schizencepahly. Isolated partial absence of the septum pellucidum is rare. Neuroimaging will help in detecting the complete or partial absence of septum pellucidum and associated anomalies. This will help in effective patient management.
